# Carotid intima-media thickness in patients with end-stage renal disease

**DOI:** 10.4103/0971-4065.50674

**Published:** 2009-01

**Authors:** K. Sunil Kumar, A. Y. Lakshmi, P. V. L. N. Srinivasa Rao, G. C. Das, V. Siva Kumar

**Affiliations:** Department of Nephrology, Sri Venkateswara Institute of Medical Sciences, Tirupati, India; 1 Department of Radiology, Sri Venkateswara Institute of Medical Sciences, Tirupati, India; 2 Department of Biochemistry, Sri Venkateswara Institute of Medical Sciences, Tirupati, India

**Keywords:** Carotid intima-media thickness, end-stage renal disease, atherosclerosis

## Abstract

Accelerated atherosclerosis and cardiovascular disease are major causes of morbidity and mortality in patients of end-stage renal disease. Carotid intima media thickness is taken as a useful surrogate marker of atherosclerosis. Thirty end-stage renal disease (ESRD) patients were subjected to ultrasonography to study CIMT before the initiation of dialysis. CIMT was found to be higher in ESRD patients than in controls. Levels of a serum marker of oxidative stress were also found to be higher in patients than in the controls. CIMT is an easy, noninvasive, reproducible, and cost-effective investigation in patients with chronic renal failure.

## Introduction

Accelerated atherosclerosis and cardiovascular disease have been recognized for many years as major causes of morbidity and mortality in patients of end-stage renal disease (ESRD).[1] Measurement of CIMT, a method initially described by Pignoli *et al.*,[2] is being increasingly used to assess atherosclerosis, to monitor the progression and the effects of treatment, and also, as a surrogate end point in clinical trials.

The IMT corresponds to the intima-media complex, which comprises of endothelial cells, connective tissue, and smooth muscle, and is the site of lipid deposition in plaque formation. The average and maximum CIMT in healthy adults were 0.67 and 0.70 mm respectively.[3–6]

The aim of this study was to examine the frequency of CIMT in ESRD patients at the time of initiation of dialysis.

## Materials and Methods

This was a prospective study that sought to assess carotid intima-media thickness in thirty ESRD patients at the time of initiation of dialysis in our unit. The intima-media thickness (IMT) of both carotid arteries was investigated ultrasonologically with an Aloka Prosound SSD 5000 using a 7.5MHz high-resolution linear probe. IMT was defined as a low level echo grey band that does not project into the arterial lumen and was measured at the diastolic phase as the distance between the leading edge of the first and second echogenic lines.[7] CIMT was measured on the longitudinal views of the far wall of the distal segment of the common carotid artery, the carotid bifurcation, and the initial tract of the internal carotid artery on both sides. Measurements were performed 0.5, 1, and 2 cm below and above the bifurcation (six measurements on each side) in a plaque-free arterial segment.[7] Each measurement is an average of three measurements and all measurements were done by a single observer. The highest measurement of the obtained values was taken as CIMT. The mean values of CIMT thickness between patients and matched controls were compared statistically by using Student's t-test.

## Results

Carotid intima-medial thickness (CIMT) was measured in 30 ESRD patients at the time of initiation of dialysis in our unit. Twenty-six of these 30 patients were men and four were women; the mean age was 43.4 (15–66) years. Eight were smokers and three were alcoholics; diabetes was observed in 12 and hypertension in 27.

**Figure 1:**
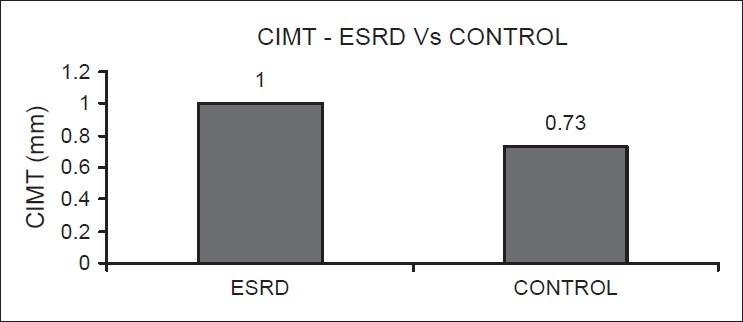
Showing the mean values of carotid intima-media thickness in end-stage renal disease patients and controls (*P* = 0.0036)

Results (mean values) of blood chemistry examination were as follows: hemoglobin, 7.34±2 g/dL; blood urea, 217±63 mg/dL; serum creatinine, 12.7±5.7 mg/ dL; serum albumin, 3.2±0.4 g/dL, serum cholesterol 141±51 mg/dL; serum triglycerides, 110±60 mg/dL, and serum calcium and phosphorous product, 53±25 mg^2^/dL^2^. The mean Body Mass Index was 1.58±0.17. The mean value of serum malondialdehyde levels, a marker of oxidative stress, was 2.29±2 μmol/L(controls: 0.82±0.28). The mean CIMT value in ESRD patients was 1.0 mm as compared to 0.73 mm in the controls [Figure 1].

## Discussion

Carotid intima-media thickness (CIMT) is a noninvasive marker that suggests the presence of generalized atherosclerosis and is also a good indicator of the presence of coronary artery disease.[1,4] The morbidity and mortality arising from atherosclerotic vascular disease is greatly increased in ESRD patients. In addition to traditional risk factors such as age, obesity, hypertension, hyperglycemia, and hyperlipidemia, uremia-related risk factors such as hemodynamic overload, anemia, malnutrition, increased oxidative stress, and hyperhomocystinemia have been cited as the causative factors for accelerated atherosclerosis and increased vascular disease in patients with chronic renal failure.[1] Hence, CIMT measurement by ultrasonography is often used as a noninvasive modality to study vascular atherosclerotic disease in patients with chronic renal failure.

In our observation of thirty ESRD patients, CIMT was found be higher than in healthy controls (*P* = 0.0036). The mean serum MDA level, a marker of oxidative stress, was found to be significantly high in the patient group in comparison to the controls. The mean values of serum cholesterol, serum triglycerides, and serum calcium and phosphorous levels were within normal limits. Serum paratharmone and homocystiene levels were not measured. A detailed evaluation of coronary artery disease was not done.

## Conclusion

The CIMT, an early and noninvasive marker of generalized atherosclerosis, was observed to be significantly high in our ESRD patients. We found the measurement of CIMT with ultrasonography to be an easy, noninvasive, reproducible, and cost-effective investigation in patients with chronic renal failure.
